# A single TRPV1 amino acid controls species sensitivity to capsaicin

**DOI:** 10.1038/s41598-020-64584-2

**Published:** 2020-05-15

**Authors:** Ying Chu, Bruce E. Cohen, Huai-hu Chuang

**Affiliations:** 1 Institute of Molecular Biology, Academia Sinica, Taipei, 11529 Taiwan; 2 The Molecular Foundry, Lawrence Berkeley National Laboratory, Berkeley, CA 94720 USA

**Keywords:** Biochemistry, Cell biology, Chemical biology, Molecular biology, Neuroscience, Physiology

## Abstract

Chili peppers produce capsaicin (a vanilloid) that activates the transient receptor potential cation channel subfamily V member 1 (TRPV1) on sensory neurons to alter their membrane potential and induce pain. To identify residues responsible for differential TRPV1 capsaicin sensitivity among species, we used intracellular Ca^2+^ imaging to characterize chimeras composed of capsaicin-sensitive rat TRPV1 (rTRPV1) and capsaicin-insensitive chicken TRPV1 (cTRPV1) exposed to a series of capsaicinoids. We found that chimeras containing rat E570-V686 swapped into chicken receptors displayed capsaicin sensitivity, and that simply changing the alanine at position 578 in the S4-S5 helix of the chicken receptor to a glutamic acid was sufficient to endow it with capsaicin sensitivity in the micromolar range. Moreover, introduction of lysine, glutamine or proline at residue A578 also elicited capsaicin sensitivity in cTRPV1. Similarly, replacing corresponding rTRPV1 residue E570 with lysine or glutamine retained capsaicin sensitivity. The hydrophilic capsaicin analog Cap-EA activated a cTRPV1-A578E mutant, suggesting that A578 may participate in vanilloid binding. The hydrophilic vanilloid agonist zingerone did not activate any A578 mutants with capsaicin sensitivity, suggesting that the vanilloid group alone is not sufficient for receptor activation. Our study demonstrates that a subtle modification of TRPV1 in different species globally alters capsaicin responses.

## Introduction

Pain occurs following intense or damaging stimulation of nociceptors, which are peripheral sensory neurons that express the transient receptor potential cation channel subfamily V member 1 (TRPV1). TRPV1 functions as an integrator of physical and chemical noxious stimuli. When activated by noxious chemicals, heat (>43 °C) or acid (pH ≦ 5.9), TRPV1 permits cation influx to elicit membrane currents^1–3^. TRPV1 enables these primary afferent neurons to distinguish noxious environmental signals from innocuous events, thereby protecting organisms against further injury. A key exogenous ligand for studying environmental stimuli of TRPV1 is capsaicin, a small lipophilic molecule in chili peppers that induces sensations of heat. Capsaicin and protons both lower the temperature threshold of TRPV1 activation, so that it is activated at room temperature^4^. Capsaicin can permeate the plasma membrane and bind to the intracellular face of TRPV1^5^, and it has been reported to pass into the cytosol to activate TRPV1 channels on the endoplasmic reticulum (ER)^6^.

Besides capsaicin, TRPV1 may also be activated by plant-derived pungent compounds, and particularly vanilloid-containing compounds such as resiniferatoxin^7^, piperine^8^, and zingerone^9,10^. TRPV1 channels cloned from different species share a conserved function as integrators of noxious stimuli, but sensitivity to pungent chemicals varies among orthologs. Rodent and human TRPV1 channels have high vanilloid sensitivities, exhibiting EC_50_ values in the nanomolar range^11–13^. In contrast, frog (*Xenopus tropicalis*) and rabbit (*Oryctolagus cuniculus*) TRPV1 channels are less sensitive to capsaicin^14,15^, and chicken TRPV1 shows the least sensitivity^16^. Establishing the pharmacological characteristics of TRPV1 may provide a means to efficiently target and activate or inhibit the channel, representing a strategy for designing analgesic drugs^17–19^.

A previous study revealed that specific mutations of the Y511 or S512 residues in the intracellular domain of TRPV1 specifically suppressed the capsaicin responsiveness of rat receptors without affecting their proton-induced current^16^. Mutating residues I550 and L547 of rabbit TRPV1 (oTRPV1) (i.e. residues equivalent to T550 and M547 in the rat receptor) enhanced capsaicin sensitivity and resiniferotoxin (RTX) binding, respectively^14,20^. Residues A561 and Y523 in frog TRPV1 (xTRPV1) (corresponding to T550 and S512 in the rat receptor) have been reported as responsible for its limited capsaicin sensitivity^15^. Moreover, residue M579 of tree shrew (*Tupaia belangeri chinensis*) TRPV1 (corresponding to T550 in the rat receptor) is responsible for its low capsaicin sensitivity, enabling the tree shrew to feed on *Piper boehmeriaefolium*^21^.

Cryogenic electron microscopy (Cryo-EM) structural studies of TRPV1^22,23^ have shown that residue E570 of rTRPV1 is close to the interface between cytosol and protein. Moreover, residues E570 and Y511 constitute the lower part of the ligand binding pocket that can accommodate RTX ligand, including its vanilloid headgroup^22,23^. Capsaicin has been described as binding in a “tail-up, head-down” orientation within vanilloid binding pockets, with the vanillyl headgroup facing the residues closest to the cytosol^24^. Studies on human TRPV1 have also identified the same amino acids as rat TRPV1 as being responsible for capsaicin sensitivity, but also found additional important residues such as L518, F591 and L670^25,26^.

Many studies have focused on capsaicin-sensitive rat TRPV1, but chicken TRPV1 has received much less attention despite it being functional as a noxious stimuli integrator sensitive to heat, oxidation and acid but not vanilloids. It remains unclear why TRPV1 channels in different species show radically different capsaicin responses. Here, we reveal the molecular mechanism underlying differential capsaicin sensitivity between chicken and rat TRPV1. To achieve this, we expressed mutant TRPV1 channels in HEK293T cells and quantified their activation by ratiometric Ca^2+^ imaging to identify specific amino acids responsible for ligand activation. Our mutagenesis study shows that changing a single residue, A578 in the chick receptor, is sufficient to confer micromolar capsaicin sensitivity on cTRPV1.

## Results

### Chimeras with rTRVP1 S5-S6 have similar capsaicin sensitivity to those with rTRPV1 S1-S4

**Figure 1:**
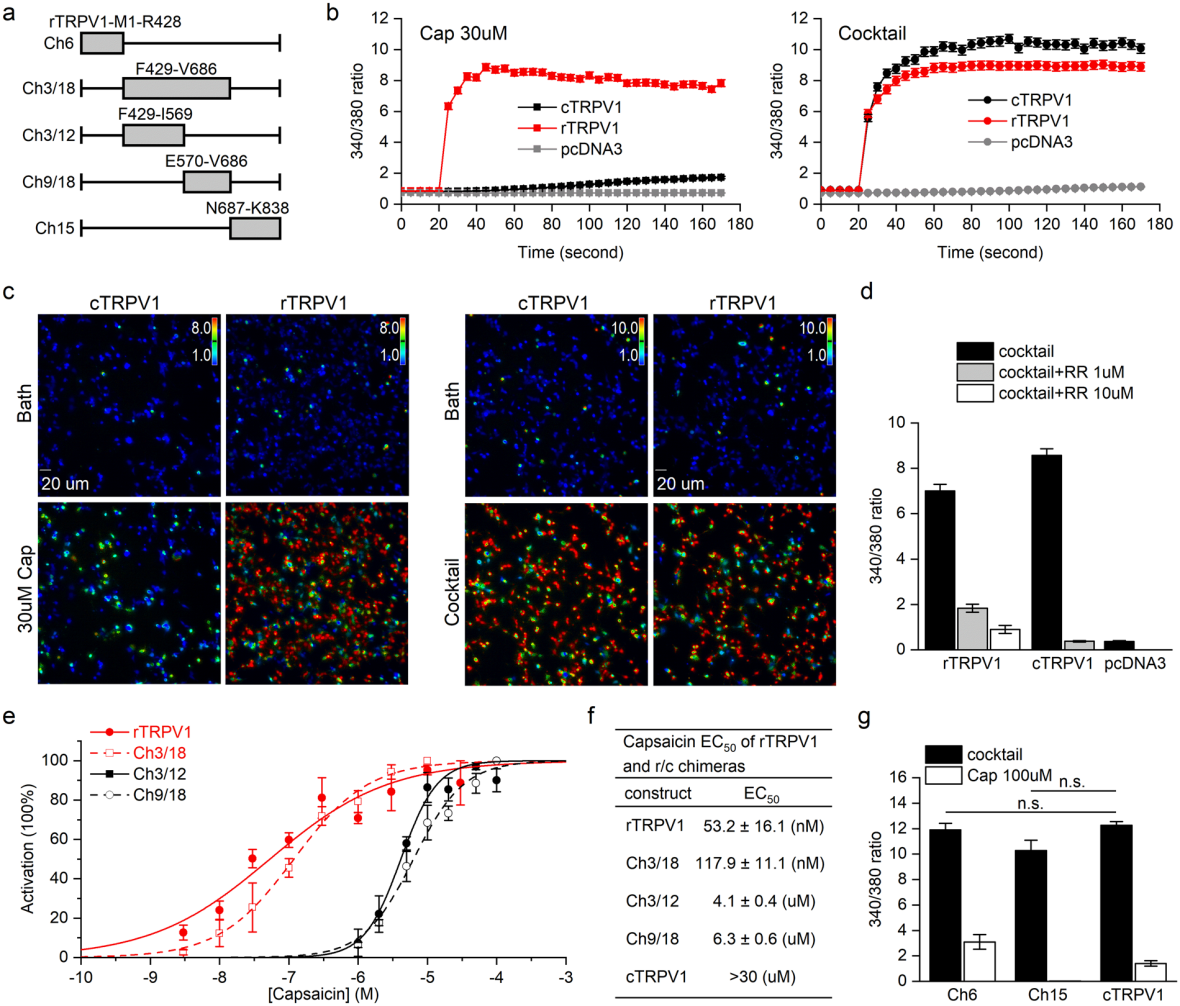
Comparison of the capsaicin sensitivity of rat-chicken TRPV1 chimeric proteins. (**a**) Structures of rat-chicken TRPV1 of chimeric proteins. The gray bars indicate the regions of rTRPV1 swapped into the cTRPV1 backbone. (**b**) Representative average calcium fluorescence signals of transient transfected HEK293T cells stimulated by capsaicin (30 μM) or cocktail. The traces present the average 340/380 ratio of cells in an entire field-of-view over time. Error bars represent SEM of the 340/380 ratio. Cells were initially incubated in bath solution and recorded for 5 time-points (25 seconds). Before the start of the 5^th^ time-point, capaicin/cocktail at 2-fold concentration was added and the increase in ratiometric signals was recorded. (**c**) Calcium imaging of rTRPV1 or cTRPV1 transient transfected HEK293T cells before (Bath) and after application of capsaicin or cocktail. Colored bars indicate ratiometric changes ranging from 1 ~ 8 (capsaicin) or 1~10 (cocktail). (**d**) Quantitative data for activation and inhibition of rat and chicken TRPV1 with cocktail solution and ruthenium red (N = 3~4). Activation of cells was calculated as the average maximum 340/380 ratio of each cell between the 5^th^-34^th^ frames after subtracting the background value. Two concentrations of ruthenium red (RR; 1 and 10 μM) were used to show its blockage effect on ions permeating rat TRPV1. 1 μM RR was sufficient to block most ions flowing through cTRPV1- and pcDNA3-transfected cells. (**e**) Dose-response curves of chimeric TRPV1 to capsaicin (N = 3), as determined by 340/380 ratio of calcium imaging data. (**f**) EC_50_ values of rTRPV1 and capsaicin-sensitive rat-chicken (r/c) chimeras to capsaicin. Since cTRPV1 exhibited a low response to 30 μM capsaicin (see Fig. 1b), the EC_50_ of cTRPV1 was estimated to be >30 μM. (**g**) Responses of chimeras with rat N- (Ch6) or C-termini (Ch15) to capsaicin and cocktail solution (N = 3). The cocktail-induced responses are not significantly different for cTRPV1/Ch15 (p = 0.08) or cTRPV1/Ch6 (p = 0.581) (Student’s t-test).

TRPV1 forms homotetrameric channels, with each subunit consisting of six α-helical transmembrane domains connected by two intracellular N- and C-terminal segments. Transmembrane domains 5 and 6 (S5-S6) constitute the central pore separated by an intervening pore loop that presumably acts as the ion selective filter. Transmembrane segments S1-S4 that surround the central pore remain static, whereas the S4-S5 linker moves to prompt opening of the TRPV1 channel^22,27^. To identify residue(s) critical for capsaicin sensitivity in TRPV1, we first analyzed rat-chicken TRPV1 chimeras (Fig. 1a and Materials and Methods) to identify regions important for the capsaicin sensitivity of the rat receptor. We used ratiometric Ca^2+^ imaging^28^ with Fura-2 to quantify increased intracellular Ca^2+^ due to TRPV1 activation. Capsaicin elicited Ca^2+^ signals in rTRPV1-transfected HEK293T cells. To verify the expression and function of mutated TRPV1 that responded weakly to capsaicin, we applied an agonist cocktail solution. Although how vanilloid ligands activate TRPV1 may be similar, slight differences in residue sensitivity to ligand-receptor interactions exist^29^. We added two strong vanilloids, capsaicin and RTX^7,30^, in order to ensure activation of mutated channels. Apart from these two vanilloids, our cocktail solution contained phenylarsine oxide (PAO), which functioned as a surrogate for oxidative stress to potentiate activation of chicken and rat TRPV1^31^, as well as cesium ions to maximize responses. We predicted that the agonist mixture could efficiently activate both wildtype rat and chicken TRPV1 as well as chimeras to achieve maximum channel activation. Our results show that 30 μM capsaicin activated both rTRPV1 and cTRPV1, but the response was relatively weak for cTRPV1 (Fig. 1b,c). In contrast, our cocktail solution induced similar activation of both rTRPV1 and cTRPV1 (Fig. 1b,c), and elicited extracellular Ca^2+^ entry could be blocked using 1 μM ruthenium red (Fig. 1d)^32^.

Cocktail and capsaicin solution both activated all wildtype and chimeric TRPV1 channels, indicating that TRPV1 protein expression and activity were not greatly impaired (Fig. S1). Chimera Ch3/18 possessing six transmembrane domains exhibited the highest capsaicin sensitivity among our chimeras. The EC_50_ value, represented by the 340/380 ratio, was slightly higher for Ch3/18 than wildtype rTRPV1, but was still within the several hundred nanomolar range. Chimeras Ch3/12 and Ch9/18 in which the rTRPV1 transmembrane domains possess both rat and chicken fragments generated EC_50_ values in the several micromolar range and retained capsaicin sensitivity (Fig. 1e,f and Table S1). Swapping the cTRPV1 N-terminus with that of rTRPV1 (chimera Ch6) resulted in a limited improvement in capsaicin sensitivity, whereas a C-terminus swap (chimera Ch15) (Cap 340/380 ratio = 0.02 ± 0.003, N = 3) exhibited no capsaicin sensitivity (Fig. 1g). Residues Y511 and S512^16^, and M547 and T550^14,15,21^, have been reported as contributing to capsaicin sensitivity and are located in segments S1-S4 that is encompassed by Ch3/12. We next determined the residue dictating capsaicin sensitivity in Ch9/18.

### A single mutation is sufficient to transfer capsaicin sensitivity to cTRPV1

**Figure 2:**
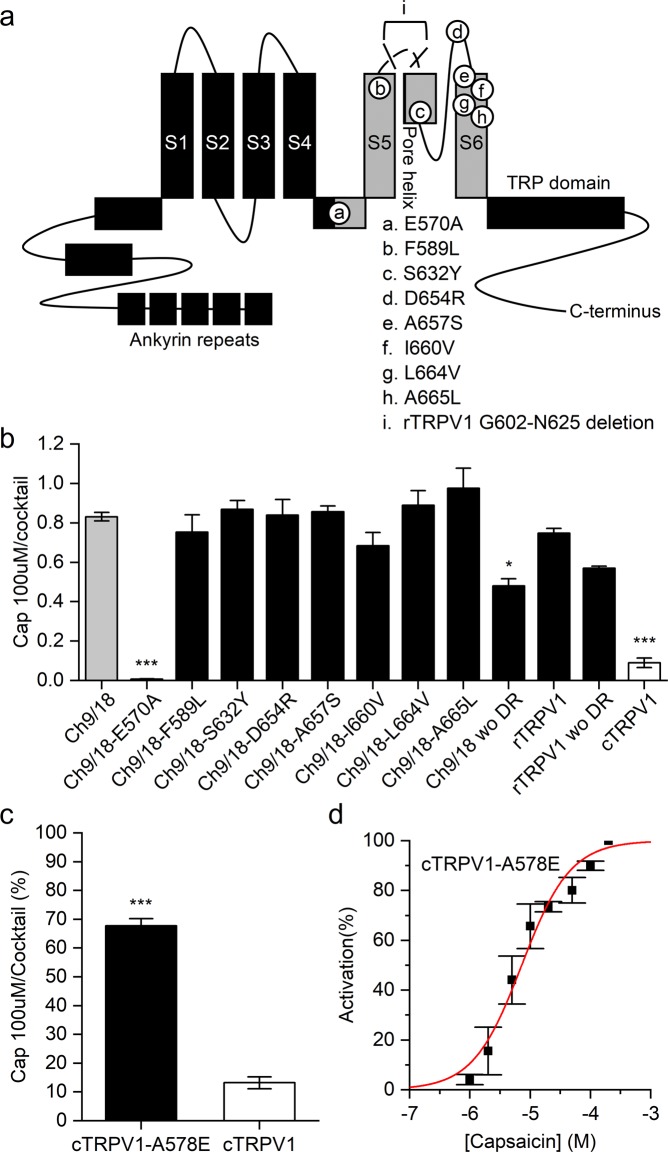
Identification of the residue endowing capsaicin sensitivity on cTRPV1. (**a**) Mutagenesis sites on chimera Ch9/18. The rTRPV1 fragment in Ch9/18 is colored gray. (**b**) The ratio of responses induced by 100 μM capsaicin (N = 3) and cocktail solution (N = 2~3) represents capsaicin-specific activation of mutant channels. Significant differences of multiple comparisons between Ch9/18 and other constructs are represented as *p < 0.05, **p < 0.01, and ***p < 0.001. The E570A mutation had the strongest impact on Ch9/18 responses to capsaicin. (**c**) Comparison of mutant cTRPV1-A578E to wildtype cTRPV1 in terms of vanilloid responsiveness (N = 3~4) (Student’s t-test, p < 0.001). Introducing the A578E mutation to cTRPV1 increased 5-fold the response of cTRPV1 to 100 μM capsaicin. (**d**) Dose-response curve of cTRPV1-A578E (N = 3), as determined by 340/380 ratio from calcium imaging data, from which the EC_50_ value for response to capsaicin (=7.2 ± 9.4 μM) was determined.

Ch9/18 differs from cTRPV1 at nine regions: residues E570A, F589L, S632Y, D654R, A657S, I660V, L664V, and A665L (all rat/chicken equivalent residues), as well as rTRPV1-G602-N625, with this last representing a region of high sequence divergence. We performed point mutagenesis of individual residues or deletion of the entire rTRPV1-G602-N625 segment on Ch9/18 to identify the residue(s) responsible for reducing capsaicin sensitivity. The equivalent sites on rTRPV1 are shown in Fig. 2a. To eliminate the effect of non-specific loss of channel function, we divided 340/380 ratio values for 100 μM capsaicin-induced activation by those for cocktail solution (Fig. S2). We found that the Ch9/18-E570A and Ch9/18 wo DR mutants (representing deletion of the G602-N625 region) exhibited significantly reduced capsaicin sensitivity relative to Ch9/18 (p < 0.001 and p < 0.05, respectively; ANOVA followed by Tukey’s Honestly Significant Difference post-hoc test for multiple comparisons). However, since deleting G602-N625 from wildtype rTRPV1 also reduces both capsaicin- and cocktail-induced activation, this region is not specifically responsible for affecting capsaicin sensitivity (Fig. 2b).

We mutated residue A578 of cTRPV1 to glutamic acid to verify the importance of residue E570 (i.e., the equivalent residue in rTRPV1) for capsaicin sensitivity. We expected that cTRPV1 with the A578E mutation would exhibit similar capsaicin sensitivity to Ch9/18 if that residue was the only one critical to endowing capsaicin sensitivity on Ch9/18. Our data shows that the A578E mutation successfully restored capsaicin sensitivity to cTRPV1 (Figs. 2c and S3) (Student’s t-test, p < 0.001), with an EC_50_ value in the micromolar range (7.2 ± 9.4 μM) (Fig. 2d) that is similar to that of Ch9/18 (6.3 ± 0.6 μM) (Fig. 1f). Previous computational modeling predicted that residue E570 participates in human and rat TRPV1 vanilloid pocket formation, and mutational experiments on that residue resulted in impaired opening of the mutant TRVP1 channel^24,33^.

### Multiple amino acid replacements of cTRPV1-A578 endow capsaicin sensitivity

**Figure 3:**
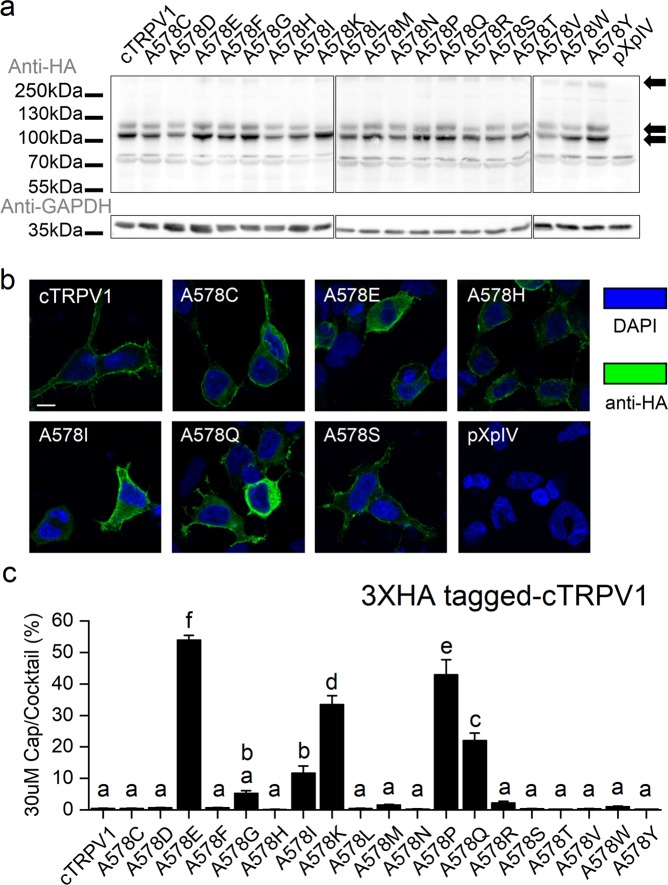
Mutational analysis of cTRPV1-A578. (**a**) Western blot of wildtype and mutant cTRPV1. Arrows point to TRPV1 aggregates, and glycosylated and non-glycosylated TRPV1 monomers. GAPDH acted as a loading control. Images of three membranes have been aligned for direct comparison between cTRPV1 wild type and mutants. Solid dividing lines delineate the boundaries of cropped photos. The non-cropped images are provided as Figure S4. The position of markers follows that of the membrane transferred with A578E mutant protein. (**b**) Immunostaining of wildtype and mutant cTRPV1 (anti-HA signal) expressed in HEK293T cells (scale bar = 10 μm, outlined by DAPI signal), showing that the proteins are located at the cell margins, representing the plasma membrane. (**c**) Vanilloid responsiveness of cTRPV1-578 mutants [30 μM capsaicin-induced activation (N = 3) normalized to cocktail-induced maximum channel opening (N = 2~3)]. Since 100 μM capsaicin treatment slightly activated even wildtype cTRPV1 (Fig. 1g), we chose 30 μM capsaicin as the standard to determine acquisition of capsaicin sensitivity among cTRPV1 mutants. Statistical significance of normalized responses of wildtype and mutant cTRPV1-578 is shown as subgroups (labeled alphabetically) at the 0.05 level based on One-way ANOVA followed by Tukey’s Honestly Significant Difference post-hoc test.

To further elucidate the mechanism by which glutamic acid could introduce capsaicin sensitivity to cTRPV1, we systematically replaced residue A578 of cTRPV1 and E570 of rTRPV1 with the other 19 amino acids. Western blotting (Figs. 3a and S4) and protein distribution imaging (Fig. 3b) revealed that the A578E mutant exhibited a similar expression level and membrane distribution to the other amino acid mutants. In Table S2, we show that the cocktail solution effectively activated most of the cTRPV1-A578 mutants, apart from the A578D, F, G, and W mutants. Using 30 μM capsaicin treatment as a standard for capsaicin responses and adjusting for cocktail-induced channel activation (Fig. S5), we found that apart from the A578E mutant, the A578K, Q, and P mutants exhibited the greatest capsaicin sensitivity (Fig. 3c). Lack of a common property among the capsaicin-sensitive-inducing replacement amino acids suggests that there are several ways to elicit capsaicin responses by altering the A578 residue of cTRPV1.

### Similar amino acid mutations of cTRPV1-A578 and rTRPV1-E570 induce capsaicin responses

**Figure 4:**
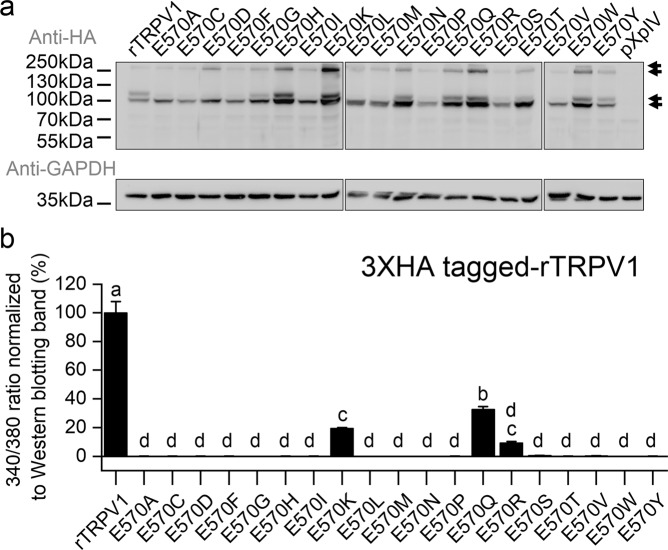
Mutational analysis of rTRPV1-E570. (**a**) Western blot of wildtype and mutant rTRPV1. Arrows point to glycosylated and non-glycosylated TRPV1 aggregates and monomers. GAPDH was used as a loading control. Images of three membranes have been aligned for direct comparison between rTRPV1 wild type and mutants. Solid dividing lines delineate the boundaries of cropped photos. The non-cropped images are provided as Figure S6. The position of markers follows that of the membrane transferred with E570A mutant protein. (**b**) Vanilloid responsiveness of wildtype and mutant rTRPV1-E570 [300 nM capsaicin-induced activation (N = 3) normalized to respective protein expression level as determined by Western blot]. Based on our results for Ch3/12 and even though the critical residue for sensing capsaicin was impaired in that chimera, we expected that the rTRPV1-E570 mutants would still respond to a high concentration of capsaicin. Therefore, we used 300 nM capsaicin to determine if the capsaicin sensitivity of rTRPV1-E570 mutants was stronger than that of Ch3/12. Statistical significance of normalized responses of wildtype and mutant rTRPV1-570 is shown as subgroups (labeled alphabetically) at the 0.05 level based on One-way ANOVA followed by Tukey’s Honestly Significant Difference post-hoc test.

Although protein expressions of all rTRPV1-E570 mutants and wildtype were similar (Figs. 4a and. S6), the cocktail solution did not activate capsaicin responses in most of the mutants (data not shown). Therefore, we normalized the capsaicin-induced response to protein level determined by Western blotting (Table S3). Western blotting revealed considerable variation in protein expression levels among the mutants (Table S3), but all exhibited expression levels similar to or greater than wildtype rTRPV1. rTRPV1 caused obvious cytotoxicity to the transfected cell line, which may explain the variation in protein amounts by killing highly-expressing cells. Notably, the E570K, and Q mutants exhibited responsiveness to 300 nM capsaicin, albeit much weaker than wildtype. However, unlike the respective cTRPV1 mutant, the rTRPV1-E570P mutant exhibited reduced capsaicin sensitivity even though protein amounts of the mutant and wildtype were similar (Figs. 4b and S7). We expected that amino acids sharing similar properties to glutamic acid and that are important for the ability of the cTRPV1-A578E mutant to sense capsaicin would also induce capsaicin responses in their respective mutants. This supposition is partially supported by similar specific amino acid mutations of rat and chicken TRPV1 inducing capsaicin sensitivity (i.e. the K and Q mutants), but a species-specific functional amino acid mutant also exists (i.e. the P mutant). The cTRPV1-A578I (Fig. 3c) and rTRPV1-E570R (Fig. 4b) mutants also seemed to induce some capsaicin sensitivity based on minor increases (~10%) in 340/380 ratios relative to maximum channel activation or wildtype rTRPV1 upon adding the agonist. We are not sure of the physiological relevance of this latter finding, and do not have further discussion.

### A hydrophilic derivative of capsaicin activates both rTRPV1 and cTRPV1-A578E

**Figure 5:**
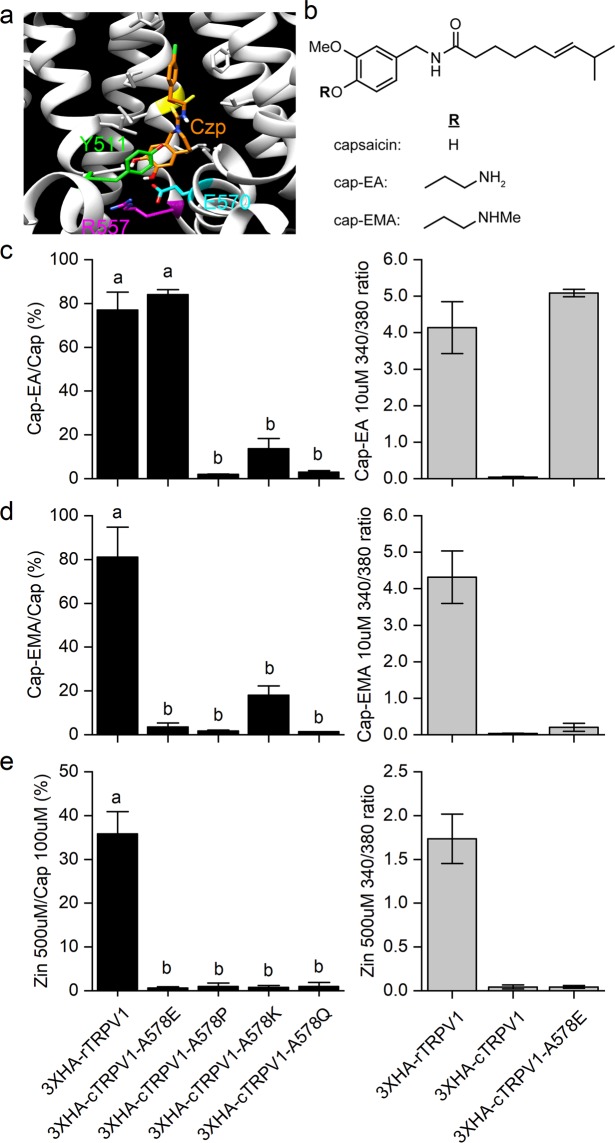
Vanilloid sensitivity of rTRPV1 and capsaicin-sensitive cTRPV1-A578 mutants. (**a**) Cryo-EM image of TRPV1 from PDB 5is0. Some critical residues close to the catechol group of the antagonist Czp are highlighted in color. (**b**) Structures of the synthetic capsaicin analogs cap-EA and cap-EMA. (**c**) Cap-EA-induced activation (10 μM) of rTRPV1 and capsaicin-sensitive cTRPV1 mutants normalized to capsaicin-induced responses (10 μM, N = 3). (**d**) Cap-EMA-induced activation (10 μM) of rTRPV1 and capsaicin-sensitive cTRPV1 mutants (N = 3) normalized to capsaicin-induced responses (10 μM, N = 3). (**e**) Zingerone-induced (500 μM) activation of rTRPV1 and capsaicin-sensitive cTRPV1 mutants normalized to capsaicin-induced responses (100 μM, N = 3). Zingerone-induced activity of rTRPV1 was 35.9 ± 5.0% that of capsaicin, so we applied a concentration lower than rTRPV1 EC50 to zingerone was used to test if A578 mutants had a strong effect on the ability of cTRPV1 to sense the vanilloid head group. In Fig. 5c–e, graphs at right show the respective 340/380 ratios for rTRPV1, cTRPV1, and cTRPV1-A578E. Letters above bars in c-e represent homogenous subsets based on One-way ANOVA followed by Tukey’s Honestly Significant Difference post-hoc test. In Fig. 5c,d, we used 10 μM capsaicinoids because it represents the saturating dose of capsaicin for rTRPV1 activation (see Fig. 1e), and it could also stimulate capsaicin-sensitive cTRPV1-A578 mutants (data not shown). Moreover, note that in Fig. 5c,d, rTRPV1 exhibits a strong response to 10 μM Cap-EA or Cap-EMA for the comparisons with cTRPV1-A578 mutants.

A cryo-EM structure of rTRPV1 bound to the antagonist capsazepine (capZ) shows that the Y511, S512, and E570 side-chains of the former are sufficiently close to the latter to allow interaction with the antagonist’s catechol group, which consists of two aromatic hydroxyl groups and differs from the hydroxyl and methoxy groups of vanilloids^23^ (Fig. 5a). To better understand how point mutation of cTRPV1 could affect capsaicin interactions with the channel, we synthesized a series of capsaicinoids with altered vanilloid groups (Fig. 5b; Li *et al*., 2011). Capsaicin has a polar vanilloid headgroup linked via an amide to an aliphatic tail, and alkylation of the vanilloid phenol oxygen has been shown to reduce, but not eliminate, channel activation. The serial chemicals were produced and most used in our study for pore-permeating capsaicin analog^34^. Capsaicinoids were prepared by alkylation of the potassium phenolate salts of capsaicin (see Materials and Methods) to generate hydrophilic *O*-ethylamine (Cap-EA) or *O*-ethylmethylamine (Cap-EMA) derivatives in which the hydroxyl group of the vanilloid was replaced. To determine how capsaicin interacts with the capsaicin-sensitive residues of cTRPV1, we tested our capsaicin-sensitive cTRPV1-578 mutants with Cap-EA or Cap-EMA (Fig. 5b). To exclude the effect of reduced analog permeability across the plasma membrane, TRPV1-expressing HEK293T cells were pre-treated with 10 μM capsaicin analog. We took advantage of Ca^2+^ imaging that uses cation-sensitive indicators to permit recording of channel activity. Pre-treatment was performed in Ca^2+^-free imaging solution, so no Ca^2+^ flow -through activated TRPV1 channels. For recordings, 10 μM analog and 2 mM Ca^2+^ in imaging solution were added to elicit extracellular Ca^2+^ entry. The solution still contains Na^+^, K^+^, and Mg^2+^, so normal cell physiology is not impaired^29,35^. For Cap-EA, the pre-incubation time was 5 minutes, whereas for Cap-EMA the pre-incubation time was 1 hour. We compared the induced responses for the analogs with those induced by 10 μM capsaicin, using the same pre-treatment and stimulation method (Figs. S8, 9). Both capsaicin analogs activated rTRPV1 but not cTRPV1. Cap-EA successfully activated Ca^2+^ current from the cTRPV1-A578E mutant (to a level not significantly different from wildtype rTRPV1) and partially activated the cTRPV1-A578K mutant (Fig. 5c). Neither the cTRPV1-A578P or Q mutants were activated by the capsaicin analogs. Cap-EMA slightly activated the cTRVP1-A578K and E mutants, but the relative increases among the A578E, K, P, and Q mutants were not significant (Fig. 5d). Thus, replacement of the hydroxide group of capsaicin with a hydrophilic side-chain drastically reduces the capacity of accessible amino acids on the receptor to activate the channel, indicating that mutants of the A578 residue of cTRPV1 have stringent structural requirements to elicit capsaicin responses.

We further tested our mutant TRPV1 receptors with the natural product zingerone from ginger (*Zingiber officinale*) to determine if its vanilloid group is sufficient to activate a channel response. Zingerone is a relatively weak vanilloid agonist that necessitates a much higher concentration than capsaicin to stimulate TRPV1 channels^36^. Zingerone lacks the aliphatic tail and acyl-amide moiety present on capsaicin. Van der Waals interactions between the aliphatic tail of capsaicin and TRPV1 are crucial for stable binding^24^. The cTRPV1 residue that likely interacts with the acyl-amide moiety of capsaicin is alanine 558. We found that 500 μM zingerone activated rTRPV1, but did not activate cTRPV1-A578E, P, K, or Q mutants (Figs. 5e and S10). The 100 μM capsaicin activated rTRPV1 and all the capsaicin sensitive mutants, but not wild-type cTRPV1. Our zingerone results demonstrate that vanilloid group alone is not sufficient to activate TRPV1 channels having theoretically-functional 578 residues. Together, our findings suggest that cTRPV1 with the A578E mutation elicits capsaicin sensitivity by endowing the previously insensitive chicken receptor with similar properties to the rat receptor (rTRPV1), and interactions of both the aliphatic tail and acyl-amide moiety with the channel are important for the cTRPV1-A578E mutant to respond to capsaicin.

## Discussion

Animals inhabiting different environments and with diverse survival strategies must evolve specific physiological adaptations. TRPV1 is critical for sensing temperature and noxious chemicals. Activation of the TRPV1 channel is the principle excitatory mechanism of chemical and thermal nociception. TRPV1 is familiar to humans as it endows us with the ability to taste spicy food and feel noxious heat. TRPV1 orthologs in vampire bats (*Desmodus rotundus*) and zebrafish (*Danio rerio*) have lower temperature thresholds than human TRPV1 and function as detectors to heat source in environments^37,38^. In contrast, ground squirrels (*Ictidomys tridecemlineatus*) and camels (*Camelus ferus*) have TRPV1 orthologs with low temperature sensitivity as an adaption to their hot environments^39^. The molecular mechanism underlying functional flexibility of TRPV1 among different animal species has been widely studied in an effort to elucidate how to control the channel, which is critical in painful sensations for humans.

The insensitivity of cTRPV1 to capsaicin has been attributed to the need for seed dispersal. Capsaicin has been reported to selectively discourage mammalian seed predators such as the packrat (*Neotoma lepida*) and cactus mouse (*Peromyscus eremicus*), but not the avian thrasher (*Toxostoma curvirostre*), with seeds consumed by the thrasher exhibiting good germination rates^40^, suggesting that directed deterrence by chili peppers may shape plant-vertebrate interactions. In general, avian TRPV1 is relatively insensitive to capsaicin. Nonivamide and resiniferatoxin, both vanilloids that occur in hot peppers, can also activate TRPV1^7,41^. Establishing how TRPV1-expressing nociceptors distinguish different strengths and types of stimulation is important to enable specific targeting of nociceptors for analgesic applications and hyperalgesia treatments, as well as to understand vanilloid-induced activation in order to generate competitive antagonists such as capsazapine, 6-iodononivamide, and 6-iodo-RTX.

Chicken TRPV1 is usually considered to be capsaicin-insensitive, although our treatment with a high concentration (100 μM) of capsaicin did trigger a limited response (Fig. 1d). The high sequence similarity between chicken and rat TRPV1^16^ suggests that acquiring capsaicin sensitivity depends on only minor sequence changes. Frog, rabbit, and zebrafish TRPV1 orthologs are all considered weak capsaicin sensors, and the respective residues corresponding to rat S512 and T550 in these three divergent species have been shown to play key roles in capsaicin sensitivity^14,15,21,38^.

In our study, we constructed chimeric rat-chicken TRPV1 channels and conducted amino acid mutagenesis to test their ability to mediate Ca^2+^ influx upon stimulation by various capsaicin analogs. Our chimeric channels encompassed four functionally different regions of TRVP1. The TRPV1 transmembrane domain is responsible for ligand sensitivity. The N- and C-termini play a role in modulating activation strength and sensitivity^31,42^. Moreover, alternative exon splicing or partial deletion of the C-terminus have been shown to generate TRPV1 channels with lower temperature thresholds^37^, whereas truncation of most of the C-terminus results in a non-functional channel^43^. Thus, the binding sites for lipophilic ligands are likely located within the transmembrane domain, and multiple studies have identified critical transmembrane residues as being responsible for vanilloid sensitivity in rTRPV1. However, since few studies had focused on cTRPV1, the possibility remained that the N and C-termini of the protein may mediate species differences. However, our results show that the chimeric channel hosting the rat transmembrane domain had obvious capsaicin sensitivity whereas chimeras harboring the rat N or C-termini did not.

We found that, within the transmembrane region, deletion of the region spanning G602-N625 impaired capsaicin-induced activation. Partial deletion of this region was previously reported to impair the heat sensitivity of mouse TRPV1, and full deletion eliminated channel current^44^. By mutating residue A578 on cTRPV1 to glutamic acid, we have demonstrated that a single point mutation can render cTRPV1 sensitive to capsaicin, and we also found that mutations to lysine (K), glutamine (Q) or proline (P) also enhanced cTRPV1 capsaicin sensitivity. This ability for site mutagenesis to endow capsaicin sensitivity is mediated by the structure of capsaicin itself, as we tested capsaicin analogs with a hydrophilic moiety on the vanilloid head and found that they exhibited decreased potency in terms of mutant TRPV1 activation relative to wildtype rTRPV1. Only the cTRPV1-A578E mutant was strongly activated by Cap-EA (ethylamine), but it was not activated by Cap-EMA (methylethylamine). The cTRPV1-A578K mutant was slightly activated by both analogs. We can confirm that the conversion was reversible on rat, as the E570A mutation drastically decreased its capsaicin-induced responses (Fig. 4). This finding supports the importance of residue E570 in rat TRPV1 for sensing capsaicinYang *et al*. (2015). previously demonstrated that E570 (residue E571 in their paper) is responsible for forming hydrogen bonds with the vanillyl head group of capsaicin, thereby pulling the S4-S5 linker to open the channel pore. This mutation not only decreases the capsaicin sensitivity of the murine channel, but also makes the channel have smaller open probability for capsaicin. Similarly, E570G mutation of rTRPV1 was previously reported to make the channel less receptive to both temperature and capsaicin stimuli^45^, supporting that this residue of rTRPV1 is critical for capsaicin binding and channel gating. Our systematic mutagenesis of rTRPV1 shows that the E570Q and K mutants also possess capsaicin sensitivity. We further tested mutant sensitivity with zingerone, another vanilloid but with a short aliphatic tail, and found that without the van der Waals interactions between the aliphatic tail and the channel binding pocket, a high concentration is required to activate even wildtype rTRPV1. None of the capsaicin-sensitive cTRPV1 mutants responded to 500 μM zingerone, supporting that the aliphatic tail of vanilloids is an important feature for activating the TRPV1 channel. These results suggest that the E570/A578 residue shares a similar mechanism in rat and chicken TRPV1 for capsaicin-mediated channel opening. The structural requirements necessary for the cTRPV-A578 mutants to sense capsaicinoids implies that this residue is involved in capsaicin binding. Although we took advantage of using a cocktail containing multiple strong TRPV1 agonists to establish if the mutant channels are functionally active, we unexpectedly encountered the problem of activating the rTRPV1 channel harboring a mutated E570 residue. This residue in segments S4-S5 is responsible for transmitting the conformational change induced by capsaicin binding to the S5-S6 pore domains. We anticipated that PAO-potentiated rTRPV1-E570 mutants would still possess some vanilloid sensitivity and be activated. The severe impairment of mutant channels to open in response to treatment with the cocktail solution (which contained PAO) shows that mutation of the E570 residue can also cause other unknown limits to channel functionality.

Our experimental approach of mutating the A578 residue of cTRPV1 critical for capsaicin-induced activation allows us to conclude that the different vanilloid sensitivity of birds and mammals is due to a single amino acid change. The alanine at residue 578 is conserved among some avian TRPV1 orthologs (e.g., duck, XM_021274874; ostrich, XM_009672965; hummingbird, XM_008495099). However, this residue is glutamic acid in *Xenopus tropicalis* and rattlesnake (*Crotalus atrox*)^46^, and glutamine in zebrafish, suggesting that loss of capsaicin sensitivity emerged in the avian lineage. Overall, our results demonstrate that capsaicin sensitivity can be endowed simply by mutating one amino acid. Apart from sensing extracellular chemical stimuli, TRPV1 also possesses multiple important biological roles in detecting temperature and participating in inflammatory reactions^47^. However, few studies have focused on the physiological impacts of mutating capsaicin-sensitive residues. A large-scale study addressing the temperature sensitivity and inflammatory responses of the mutants identified in the present study would be illuminating.

## Methods and materials

### Molecular cloning

Wild-type rat (*Rattus norvegicus*) and chicken (*Gallus gallus*) TRPV1 genes in pcDNA3 plasmid^48^ were used to construct chimeras by overlap extension PCR, swapping the rTRPV1 sequences with corresponding cTRPV1 fragments including the rTRPV1 N-terminus (M1-R428; denoted chimera Ch6), S1-S4 (F429-I569; denoted Ch3/12), S5-S6 (E570-V686; denoted Ch9-18), S1-S6 (F429-V686; denoted Ch3/18), and the C-terminus (N687-K838; denoted Ch15). For single point-mutated cTRPV1 and rTRPV1, the genes were cloned into pxpIV plasmids and linked with three HA tag (3XHA) repeats at the N-terminus for Western blotting and immunostaining. Point mutations were introduced by QuikChange mutagenesis using PfuUltra II Fusion HS DNA Polymerase (Aligent). To remove rTRPV1-G602-N625 (GKNNSLPMESTPHKCRGSACKPGN) sequences from Ch9/18 and rTRPV1 genes, the sequence was deleted by back-to-back PCR (Phusion Hot Start Flex DNA Polymerase, New England Biolab) and ligated to blunt ends (T4 DNA ligase, Thermo Scientific). Plasmids were sequenced by Genomics BioSci & Tech (Taiwan), and then transformed and amplified in DH5α competent cells (Yeastern Biotech).

### Mammalian cell culture

HEK293T cells were grown in MEM/EBSS (HyClone) medium with 10% fetal bovine serum (FBS, Gibco), and 100 U/ml penicillin and 100 μg/ml streptomycin (Lonza). The incubator was maintained at 37 °C with 5% CO_2_. The cells were seeded onto plates one day before transfection, and reached 60-90% confluency by the time for transfection. OptiMEM (Life Technology) and Avalanche-Omni Transfection Reagent (EZ Biosystems) were mixed with plasmids and added into wells with HEK293T cells. After two days, the transfected cells were prepared for Ca^2+^ imaging, immunostaining or Western blotting.

### Ratiometric Ca^2+^ imaging

The 96-well plates were coated with poly-D-lysin (0.1 mg/ml) and collagen (55 μg/ml). The transfected cells were added to 96-well plates with MEM + 5.4% FBS + penicillin/streptomycin and grown overnight. Cells were loaded with 0.02% pluronic F-127 (Life Technology) and 2 μM Fura-2 AM (Life Technology)^49,50^ for 3-5 hours in imaging solution [8.5 mM HEPES, 140 mM NaCl, 3.4 mM KCl, 1.7 mM MgCl_2_, and 1 mM CaCl_2_, pH 7.4] at 30 °C with 5% CO_2_. Solutions were replaced with the same imaging solution without Fura-2 AM before imaging. Background-subtracted, emitted fluorescence following excitation at 340 nm and 380 nm was detected using an EMCCD camera (Photometrics, Evolve) driven by Slidebook 6 digital microscopy software (Intelligent Imaging Innovations). Fluorescence data were acquired by capturing the frame rate at one frame every 5 sec with 20-50 ms exposure time to either wavelength. Over 160 cells in the recording fields were included for data analysis. Ca^2+^ imaging experiments were conducted at 22 °C, which is well below the stimulating temperature of TRPV1 (>43 °C)^51^.

Capsaicin (Pfaltz & Bauer), zingerone (Pfaltz & Bauer), and a cocktail were prepared as stock solutions in DMSO (Calbiochem). The cocktail solution used to induce maximum TRPV1 activation contained 100 μM capsaicin, 5 μM resiniferatoxin (Ascent), and 100 μM phenylarsine oxide (Alfa Aesar). The cocktail ligands were dissolved in solution that replaced 140 mM NaCl with 140 mM CsCl. Reactive chemicals or ligands were prepared as 2X concentration stocks (2-fold).

The concentration of ligands reached 1X after being applied to wells containing an equivalent amount of imaging solution (75 μl). The CsCl concentration in the cocktail solution was 70 mM after being added to imaging solutions. For pre-treatment experiments, dye-loaded cells were incubated with chemicals (1X concentration) in imaging solution without Ca^2+^, and ligand (1X concentration) with 2 mM CaCl_2_ was added during recording.

Agonists were added at the 5^th^ frame after beginning recording. The background 340/380 ratio value was calculated as the average ratio of the 0-4^th^ frames of all cells. Agonist-induced activation was calculated as the average maximum 340/380 ratios of each cell between the 5^th^-34^th^ frames after subtracting the background value. One-way ANOVA followed by Tukey’s Honestly Significant Difference post-hoc test for multiple comparisons or Student’s t-test was applied to determine statistical significance and is indicated as *P < 0.05, **P < 0.01, or ***P < 0.001. The “a, b, c…” labeling in Figs. 3–5 denotes homogenous subsets of results based on multiple comparisons of post-hoc tests at a mean significance level of 0.05. A dose-response curve was fitted to the sigmoidal dose-response equation: Y = min + (max-min)/(1 + 10^ [(LogEC_50_-X) x Hill Slope]).

### Western blotting

Transiently transfected HEK293T cells were homogenized in lysis buffer [150 mM NaCl, 50 mM Tris pH 7.5, 2 mM EDTA, 0.5% Triton X-100, 0.5% NP-40] mixed with protease inhibitor cocktail set III (EDTA-free, Calbiochem). Concentrations of protein samples were measured by BCA assay (Thermo Scientific, Pierce). Protein lysates (25 μg) mixed with 6X sample buffer with 1 mM DTT were resolved by SDS-PAGE and transferred onto PVDF. HA-tagged TRPV1 protein was identified by rabbit polyclonal primary antibody HA.11 (Covance PRB-101P, 1:2000 dilution). GAPDH was detected by rabbit polyclonal GAPDH antibody (Santa Cruz Biotechnology FL-335, 1:5000 dilution). Both antibodies were visualized by anti-rabbit IgG HRP-conjugated secondary antibody (Pierce #31430, 1:5000 dilution), and the blot images were acquired using BioSpectrum 810 (UVP). The images were analyzed using ImageJ. The HRP signal of each band was normalized to the rTRPV1 band on the same membrane in order to compare results from different days and different membranes.

### Immunostaining and microscopy imaging

Chamber slides were coated with PDL and collagen separately and left overnight. The transiently transfected HEK293T cells were seeded onto the PDL and collagen-coated chamber slides and incubated at 37 °C and 5% CO_2_ overnight. The cells were fixed with 4% paraformaldehyde in 1X DPBS, and then permeabilized with 1X DPBS + 0.5% Triton X-100. After treatment with blocking buffer [1X DPBS + 1% bovine serum albumin (BSA, *w*/*w*) + 0.5% Triton X-100] for 1 hour, the samples were incubated with primary and secondary antibodies, i.e., HA.11 Clone 16B12 monoclonal antibody (Covance MMS-101P, 1:1000) and Alexa Fluor 488 goat anti-mouse Ab (Life Technology A11001, 1:5000), respectively. The slides were stained with DAPI, mounted in SlowFade Diamond antifade (Life Technology), and sealed with coverslips. Images were acquired using an LSM780 laser scanning confocal microscope (Carl Zeiss).

### Capsaicinoid synthesis

*O*-aminoethyl capsaicin (**cap-EA**) was synthesized in a two-step procedure from capsaicin (Li *et al*., 2011). Boc-aminoethyl bromide was dissolved in 3 mL of dry THF and added to capsaicin (305 mg, 1.0 mmol), potassium *t*-butoxide (112 mg, 1.0 mmol), and 18-crown-6 (264 mg, 1.0 mmol) dissolved in 7 mL of dry THF. The reaction was stirred overnight, concentrated, and partitioned between EtOAc and saturated NH_4_Cl solution. The organics were washed well with water, then dried and concentrated to 439 mg of crude white solid. Of this latter, 150 mg (0.33 mmol) was dissolved in 1 mL CH_2_Cl_2_, and 35 μL (0.35 mmol) thiophenol and 0.5 mL of TFA were added. After 30 min, the solvent was evaporated and the residue was purified by HPLC on a semi-preparative 18 column, eluting over 50 min with a linear gradient of 20 to 70% CH_3_CN in 50 mM NH_4_OAc pH 4.5. The cap-EA peak eluted at 40 min (52% CH_3_CN) and free capsaicin at 55 min (65% CH_3_CN). Fractions were lyophilized to a white powder (81 mg, 54%). ^1^H NMR (DMSO-*d*_*6*_, 400 MHz) showed additional peaks relative to capsaicin at 4.66 (t, 2 H) and 3.39 (t, 2 H). MS calcd for C_20_H_33_N_2_O_3_^+^ (MH^+^): 349.2. Found: 349.2.

To generate *O*-(*N*-methylaminoethyl) capsaicin (**cap-EMA**), the acetate salt of cap-EA (20 mg, 49 μmol) was dissolved in 1 mL of dry DMF with triethylamine (7 μL, 50 μmol) and cooled to 4 °C. Iodomethane (2 μL, 33 μmol) was dissolved separately in 1 mL of dry DMF and added dropwise to the reaction over 10 min. The reaction was stirred for 2 h at 4 °C and then purified by reverse phase HPLC as described above, with the product eluting at 43 min (55% CH_3_CN). The fractions were lyophilized to white powders. Based on ^1^H NMR (DMSO-*d*_*6*_, 400 MHz), cap-EMA showed additional peaks relative to capsaicin at 4.62 (t, 2H), 3.38 (t, 2H), and 2.90 (s, 3H). MS calcd for C_21_H_35_N_2_O_3_^+^ (MH^+^): 363.5. Found: 363.4.

## Supplementary information

Supplementary Information.

## Data Availability

All data needed to evaluate the conclusions in the paper are present in the paper. Additional data related to this paper may be requested from the authors.
