# Supplementary material for: A single TRPV1 amino acid controls species sensitivity to capsaicin
Source: Sci Rep. 2020 May 15;10:8038. doi: 10.1038/s41598-020-64584-2 (PMC7229161; doi:10.1038/s41598-020-64584-2)
Supplement: Supplementary file 1 — Supplementary Information. [file 41598_2020_64584_MOESM1_ESM.pdf]

A single TRPV1 amino acid controls species sensitivity to capsaicin

Ying Chu[1,*], Bruce E. Cohen[2], Huai-hu Chuang[1,†]
Affiliations:
[1] Institute of Molecular Biology, Academia Sinica, Taipei 11529, Taiwan
[2]The Molecular Foundry, Lawrence Berkeley National Laboratory, Berkeley, CA, 94720, USA

*To whom correspondence should be addressed: ying0918@gate.sinica.edu.tw
†The original corresponding author, Dr. Huai-hu Chuang, passed away on 24th January during the revision process of this manuscript

Supplementary Information

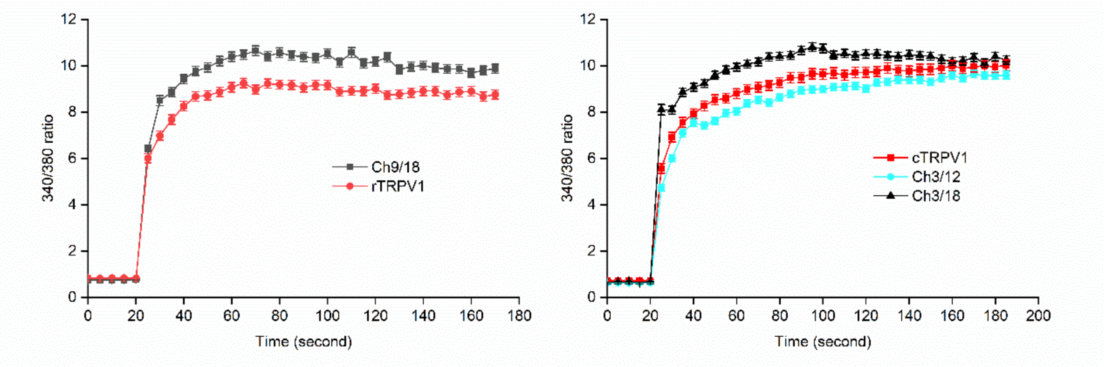

Figure S1. Comparison of average calcium fluorescence signals of HEK293T cells transfected with chimeric or wild-type TRPV1 for their response to cocktail solution. Traces in each plot represent recordings performed on the same day. Ch9/18 exhibited a moderately stronger response to cocktail solution than rTRPV1. Ch3/12 and Ch3/18 exhibited similar 340/380 ratio increments in response to cocktail solution compared to cTRPV1. Error bars represent SEM of the 340/380 ratio.

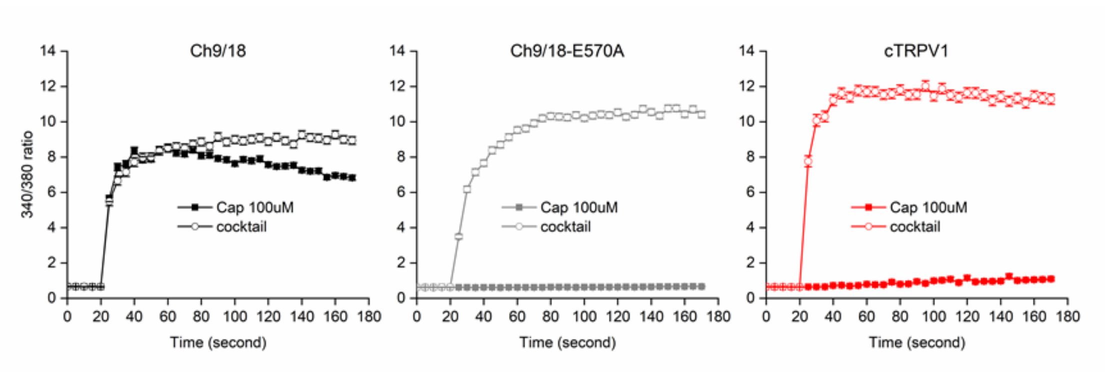

Figure S2. Representative average calcium fluorescence signals of HEK293T cells

transfected with Ch9/18, Ch9/18-E570A or cTRPV1 and stimulated by capsaicin (100 µM) or cocktail. Error bars represent SEM of the 340/380 rati

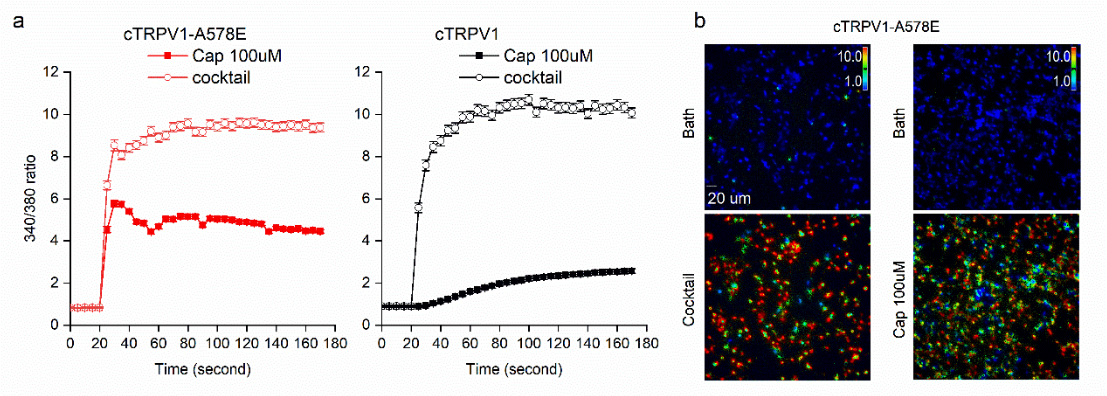

Figure S3. (a) Representative average calcium fluorescence signals of HEK293T cells expressing cTRPV1-A578E or cTRPV1 and stimulated by capsaicin (100 µM) or cocktail. Error bars represent SEM of the 340/380 ratio. (b) Images showing increased 340/380 signal from HEK293T cells expressing cTRPV1-A578E in response to cocktail or 100 µM capsaicin.

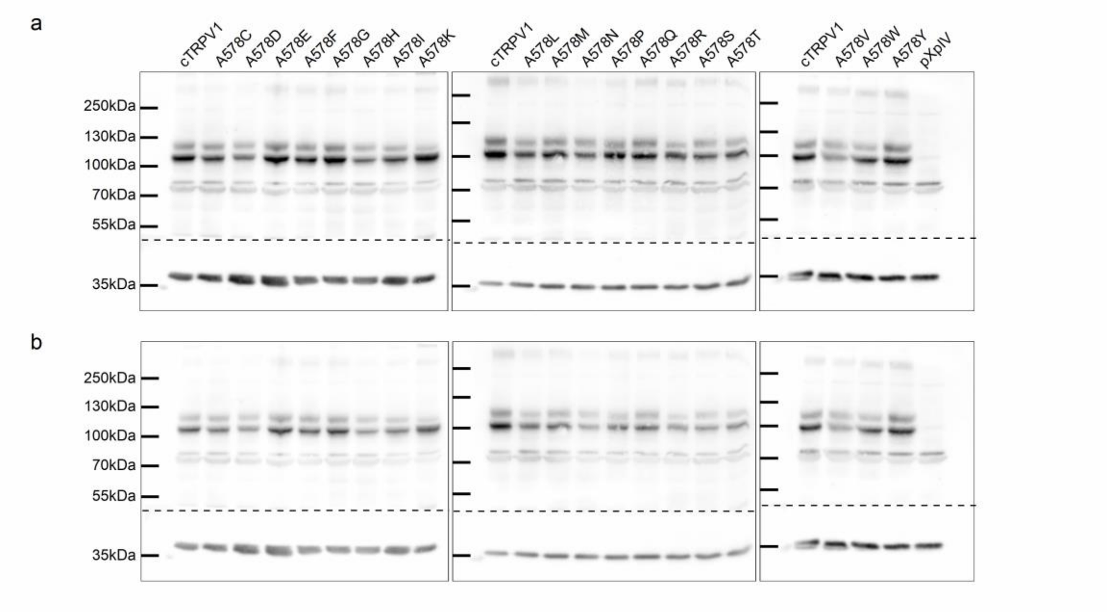

Figure S4. (a) The non-cropped PVDF membranes of the Western blot shown in Figure 3. After transferring the proteins onto PVDF, the membranes were cut into two parts at the position below 55 kDa. The two membranes were treated separately with anti-HA primary antibody and anti-GAPDH primary antibody. The anti-HA and anti-GAPDH membranes were then placed back together and treated with HRP substrate for imaging. The dashed lines represent the boundaries of the two membrane parts. HA-tagged wild-type cTRPV1 protein collected from the same batch of transfected cells was loaded in the three different SDS-PAGE as a control for comparisons with

the cTRPV1-A578 mutants on the same membrane. The bands on the left of each image represent the positions of pre-stain markers on each membrane. For Figure 3, the TRPV1 and GAPDH bands on different membranes were aligned and the marker positions of the blot with A578E were used in the combined figure. (b) The non-cropped PVDF membranes of the Western blot shown in Figure 4, but with a shorter exposure time.

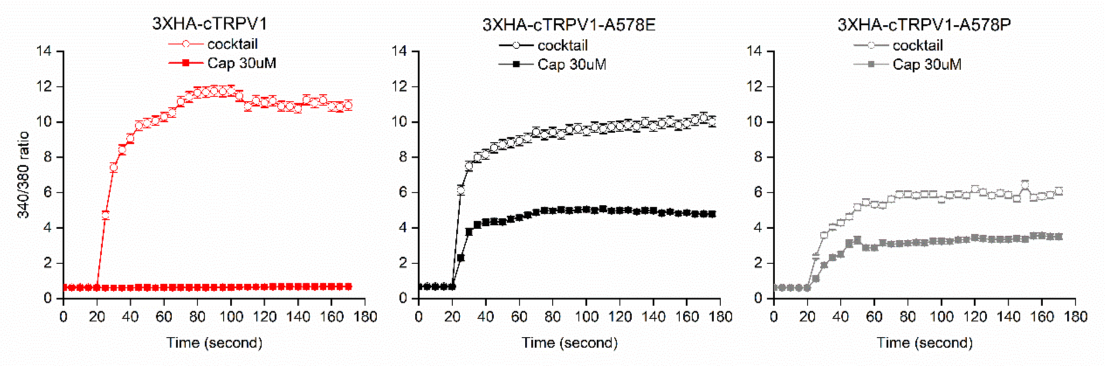

Figure S5. Selected traces of average calcium fluorescence signals of HEK293T cells expressing 3XHA-cTRPV1 with the A578E or A578P mutation in response to cocktail or 30 μM capsaicin. Error bars represent SEM of the 340/380 ratio.

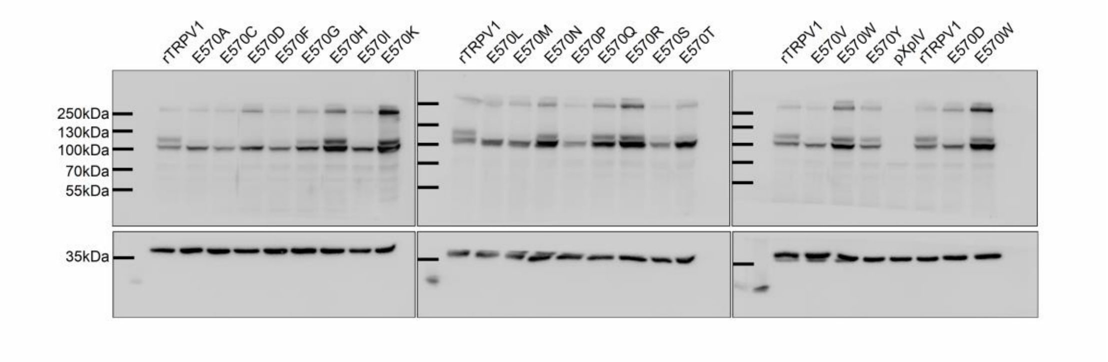

Figure S6. The non-cropped PVDF membrane of the Western blot shown in Figure 4. As for the chicken TRPV1 Western blotting approach described in Figure S6, the transferred membranes were cut into two parts and treated separately with anti-HA primary antibody and anti-GAPDH primary antibody. HA-tagged wild-type rTRPV1 protein collected from the same batch of transfected cells was loaded in the three different SDS-PAGE as a control for comparisons with the rTRPV1-E570 mutants on the same membrane. The bands on the left of each image represent the positions of pre-stain markers on each membrane. The HA-tagged and GAPDH-tagged membrane sections were not placed back together for imaging, so the images are shown separately. Secondary repeats of rTRPV1 with E570D or E570W were carried out on the same day (two rightmost lanes in the photo at right).

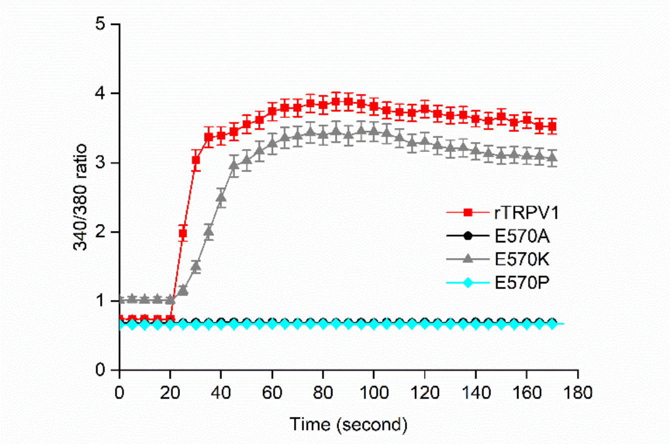

Figure S7. Selected traces of average calcium fluorescence signals of HEK293T cells expressing 3XHA-rTRPV1 with the E570A, E570K or E570P mutations in response to 300 nM capsaicin. Error bars represent SEM of the 340/380 ratio.

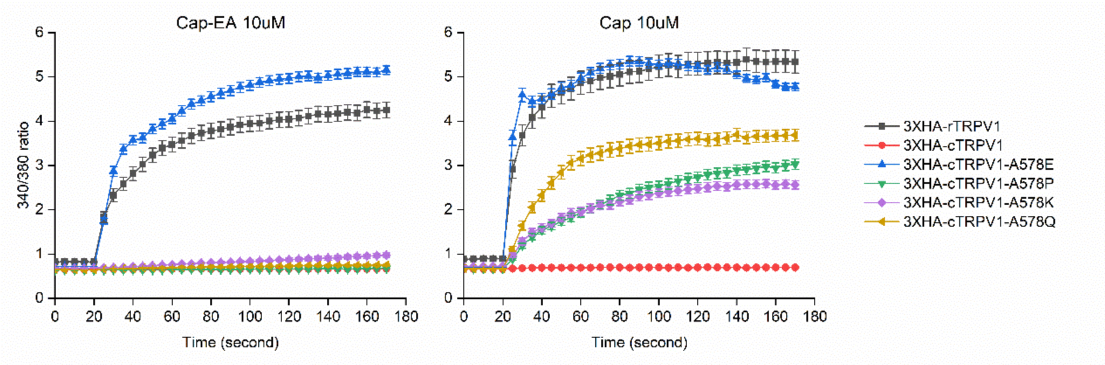

Figure S8. Average calcium fluorescence signal traces of HEK293T cells expressing 3XHA-rTRPV1 or capsaicin-sensitive 3XHA-cTRPV1 A578 mutants in response to Cap-EA (10 μM, left) or capsaicin (10 μM, right). Error bars represent SEM of the 340/380 ratio.

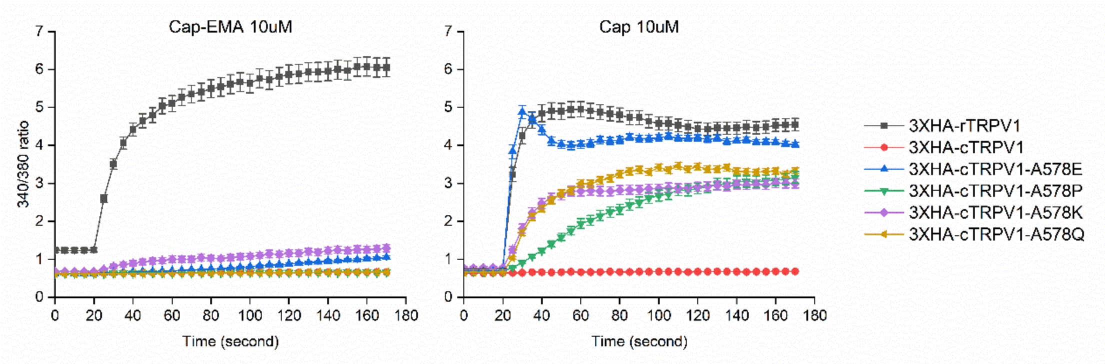

Figure S9. Average calcium fluorescence signal traces of HEK293T cells expressing 3XHA-rTRPV1 or capsaicin-sensitive 3XHA-cTRPV1 A578 mutants in response to Cap-EMA (10 μM, left) or capsaicin (10 μM, right). Error bars represent SEM of the 340/380 ratio.

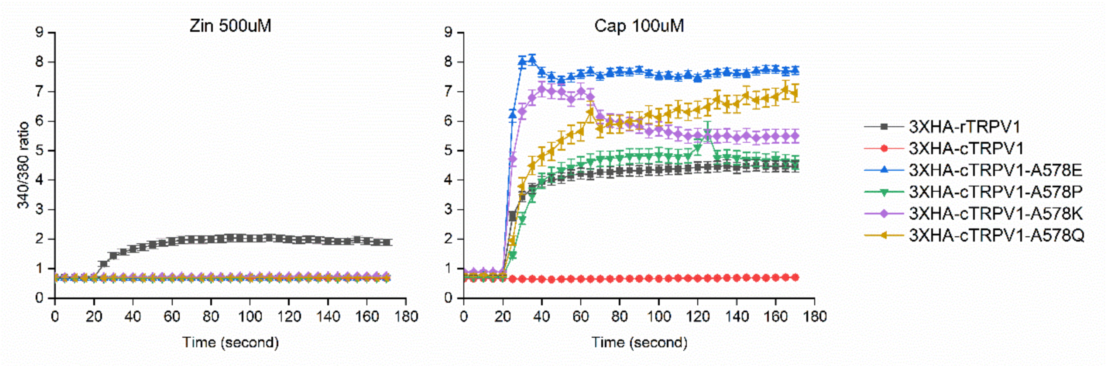

Figure S10. Average calcium fluorescence signal traces of HEK293T cells expressing 3XHA-rTRPV1 or capsaicin-sensitive 3XHA-cTRPV1 A578 mutants in response to zingerone (500 µM, left) or capsaicin (10 µM, right). Error bars represent SEM of the 340/380 ratio.

Table S1. Statistical comparison of EC_{50} values from rat-chicken chimeras and rTRPV1

	rTRPV1	Ch3/18	Ch3/12	Ch9/18
EC_{50}	53.2±16.1 (nM)	117.9±11.1 (nM)	4.1±0.4 (µM)	6.3±0.6 (µM)
*P=	-	0.018762	0.000384	0.000318

* Independent-sample two-tailed *t* tests using the EC_{50} and standard error values for each construct.

Table S2. Activation of wildtype and mutant cTRPV1 induced by cocktail solution and capsaicin, based on 340/380 ratio.		
Gene	Maximum 340/380 ratio-background (N=1)	
	Cocktail	Cap (30 μM)
3XHA-cTRPV1	12.03	0.08
3XHA-cTRPV1-A578C	11.72	0.02
3XHA-cTRPV1-A578D	3.44	0.03
3XHA-cTRPV1-A578E	11.29	5.77
3XHA-cTRPV1-A578F	2.41	0.02
3XHA-cTRPV1-A578G	2.68	0.17
3XHA-cTRPV1-A578H	14.14	0.02
3XHA-cTRPV1-A578I	11.46	0.76
3XHA-cTRPV1-A578K	12.60	4.36
3XHA-cTRPV1-A578L	8.23	0.03
3XHA-cTRPV1-A578M	11.34	0.15
3XHA-cTRPV1-A578N	7.42	0.02
3XHA-cTRPV1-A578P	6.51	3.84
3XHA-cTRPV1-A578Q	13.05	2.93
3XHA-cTRPV1-A578R	13.34	0.17
3XHA-cTRPV1-A578S	13.98	0.06
3XHA-cTRPV1-A578T	14.62	0.02
3XHA-cTRPV1-A578V	10.81	0.04
3XHA-cTRPV1-A578W	2.21	0.02
3XHA-cTRPV1-A578Y	9.17	0.02

Table S3. Quantitative data on Western blot band intensities of wildtype and mutant rTRPV1.

	Western 1	Western 2	Western 3	Average	s.e.m
WT	1	1	1	1	0
A	0.52	1.01	0.87	0.80	0.15
C	1.04	0.61	0.74	0.80	0.13
D	1.01	1.86	1.36	1.41	0.25
F	1.10	1.09	0.58	0.92	0.17
G	1.30	2.46	0.98	1.58	0.45
H	1.16	6.19	1.54	2.96	1.62
I	0.21	2.15	0.50	0.95	0.61
K	0.99	7.59	1.77	3.45	2.08
L	2.02	1.09	0.77	1.29	0.37
M	1.86	1.17	0.68	1.24	0.34
N	4.39	2.78	1.13	2.77	0.94
P	1.23	0.72	0.41	0.78	0.24
Q	6.38	2.61	0.95	3.31	1.61
R	5.07	3.65	1.51	3.41	1.03
S	0.95	0.92	0.77	0.88	0.06
T	1.61	2.27	0.83	1.57	0.42
V	1.59	0.68	0.53	0.93	0.33
W	2.61	2.80	1.53	2.31	0.39
Y	3.43	1.23	0.60	1.75	0.86
